# A Plant Based Modified Biostimulant (Copper Chlorophyllin), Mediates Defense Response in *Arabidopsis thaliana* under Salinity Stress

**DOI:** 10.3390/plants10040625

**Published:** 2021-03-25

**Authors:** Md Tariqul Islam, Wenzi Ckurshumova, Michael Fefer, Jun Liu, Wakar Uddin, Cristina Rosa

**Affiliations:** 1 Department of Plant Pathology and Environmental Microbiology, The Pennsylvania State University, University Park, PA 16802, USA; wxu2@psu.edu (W.U.); czr2@psu.edu (C.R.); 2 Suncor AgroScience, 2489 North Sheridan Way, Mississauga, ON L5K 1A8, Canada; wckurshumova@suncor.com (W.C.); mfefer@suncor.com (M.F.); juliu@suncor.com (J.L.)

**Keywords:** biostimulant, copper chlorophyllin, ROS, salinity stress, RNA-Seq

## Abstract

To date, managing salinity stress in agriculture relies heavily on development of salt tolerant plant varieties, a time-consuming process particularly challenging for many crops. Plant based biostimulants (PBs) that enhance plant defenses under stress can potentially address this drawback, as they are not crop specific and are easy to apply in the field. Unfortunately, limited knowledge about their modes of action makes it harder to utilize them on a broader scale. Understanding how PBs enhance plant defenses at cellular and molecular levels, is a prerequisite for the development of sustainable management practices utilizing biostimulants to improve crop health. In this study we elucidated the protective mechanism of copper chlorophyllin (Cu-chl), a PB, under salinity stress. Our results indicate that Cu-chl exerts protective effects primarily by decreasing oxidative stress through modulating cellular H_2_O_2_ levels. Cu-chl treated plants increased tolerance to oxidative stress imposed by an herbicide, methyl viologen dichloride hydrate as well, suggesting a protective role against various sources of reactive oxygen species (ROS). RNA-Seq analysis of Cu-chl treated *Arabidopsis thaliana* seedlings subjected to salt stress identified genes involved in ROS detoxification, and cellular growth.

## 1. Introduction

The beginning of the 21st century is manifested by continual decline of arable land and yield per capita in part due to climate change associated abiotic and biotic stresses [1]. For instance, environmental stresses like salinity, drought, cold, heat and heavy metal can cause more than 50% yield losses [2,3]. Among these stresses, salinity is one of the leading causes of crop yield reduction [4,5]. According to the Food and Agriculture Organization (FAO), salinity affects more than 30% of the irrigated land area worldwide, resulting in a monetary loss of 27.3 billion USD per year [6,7,8]. This is a ubiquitous issue and currently no continent is completely free from soil salinity [9]. It is speculated that soil salinity will increase in future climate change scenarios because of the rise of sea level and temperature, which will inevitably lead to increased evaporation and further salinization [9]. It is estimated that by 2050 50% of arable land will be impacted by salinity [10,11,12].

Salinity stress negatively influences seed germination, plant growth, physiology, yield and it can cause plant death under severe conditions [2]. At the onset of the stress, salt solutes cause accumulation of high concentration of rhizospheric ions (mainly Na+ and Cl−), thus reducing water uptake through the roots [13,14]. This consequently leads to depletion in water potential and osmotic imbalance, while at the same time excessive amounts of salt enter the plant’s transpiration system [8,12,13]. Therefore, salt stress affects plant performance in two ways, either as an inhibitor of water uptake (osmotic effect) or as an accumulator of ions, with subsequent toxic effects [14]. Additional consequences include the closure of stomata, which limits CO_2_ uptake in leaf tissues and consequently reduces carbon fixation and assimilation [15]. As a result, the photosynthesis rate and carbohydrate production are reduced, negatively impacting plant growth and yield [8]. Another major consequence of salt stress is triggering rapid accumulation of reactive oxygen species (ROS) [16,17,18,19].

ROS comprises of both free radicals (hydroxyl radical: •OH, superoxide radicals: O_2_•^–^, perhydroxy radical and alkoxy radicals: RO•) and non-radicals (hydrogen peroxide: H_2_O_2_, and singlet oxygen species: _1_O_2_). Plants generate ROS as by-products of normal cellular activity during electron transport and during stress, H_2_O_2_ in particular has been established as a signaling molecule that can trigger specific signal transduction pathways [20,21]. Under steady state conditions, the rates of H_2_O_2_ production and removal are in balance as endogenous antioxidant defense systems protect cellular homeostasis from its toxic effects [22]. Recent evidence indicates that ROS homeostasis can help in plant vegetative development [21]. For instance, in Arabidopsis H_2_O_2_ has been found to accumulate in the elongation zone of the meristem, contributing to cell differentiation [23]. ROS as signaling molecules are involved in regulating seed germination through GA and/or ABA signaling in Arabidopsis [24]. During abiotic stresses, ROS trigger signal transduction pathways in response to those stresses, resulting in environmental adaptation [21]. However, they can also increase dramatically leading to physiological and metabolic changes and to damage in plants [21]. For instance, while pulses of ROS produced by respiratory burst oxidase homologues (RBOHs) in response to stress can propagate ROS signals to prime and acclimate plants to stress [25], accumulation of ROS can disturb the cellular balance [26] and lead to, damage of DNA and proteins, lipid peroxidation and cell death in most extreme cases [27]. To control ROS concentrations in the cell and counteract stress, plants mobilize various antioxidants and ROS degrading enzymes. [22]. The antioxidant systems involve both enzymatic and non-enzymatic H_2_O_2_ scavengers. Enzymes, such as catalase (CAT), ascorbate peroxidase (APX), gluthathione S-Transferase (GST), glutathione peroxidase (GPX), glutathione reductase (GR), type III peroxidases and peroxyredoxin (Prx); and non-enzymatic compounds, like ascorbate (AsA), glutathione (GSH), α-tocopherol and flavonoids, are all involved in regulating cellular H_2_O_2_ concentrations [28].

The antioxidant mediated defense capacity may vary among plant species and genotypes, and depend on specific types of stresses and their duration [29]. Exogenous application of various chemical compounds (phytohormones, polyamines, melatonin, epigenetic inhibitors, etc.) has been shown to enhance abiotic stress tolerance, including salinity, in many plant species [22]. A relatively new addition to this list is the exogenous application of plant biostimulants (PBs). These are the often modified substances of natural origin or microorganisms that in minute quantities can promote plant growth and development through activation of the plant’s own metabolic and defense mechanisms [30]. When exposed to abiotic stresses, plants pretreated (primed) with these chemical compounds have been shown to have lower ROS accumulation and be more tolerant to oxidative stress. This is a result of plant’s enhanced ROS detoxifying/scavenging capacity which correlates with increased transcript levels of both enzymatic and non-enzymatic components of the antioxidant system [31]. Though PBs have been reported to reduce the adverse effect of stress through the activation of conserved protective pathways, their mode of protection is often poorly characterized [32,33].

Copper chlorophyllin (Cu-chl), a semi synthetic water-soluble chlorophyll derivative, has been shown to serve as oxidative stress reducing agent in mammalian cells through its presumed strong antioxidant activity [34,35,36]. More recently, the protective role of Cu-chl against drought stress in tomato has also been reported, where foliar application of Cu-chl–containing products increased leaf antioxidant enzymes activity, as well as glutathione (GSH) content [37]. However, the molecular pathways through which Cu-chl exerts its activity remain currently not understood. Here, we provide new insights into the biological function of Cu-chl in improving tolerance to high salinity stress in *A. thaliana*. We use RNA-Seq analysis to investigate the gene regulation underlying Cu-chl’s protective effect. We report that the application of exogenous Cu-chl results in upregulation of several classes of ROS detoxifying genes and genes previously involved in stress protection.

## 2. Results

### 2.1. Pretreatment with Cu-chl Reduces H_2_O_2_

Salt stress triggers the accumulation of intracellular H_2_O_2_, a signature of oxidative imbalance in plant cells [26]. To determine whether Cu-chl reduces oxidative imbalance under salt stress, we examined histochemically (diaminobenzidine (DAB) staining) the levels of H_2_O_2_ produced in salt treated *A. thaliana* seedlings primed with Cu-chl. DAB is oxidized by H_2_O_2_ typically in the presence of heme containing peroxidases, in this case horseradish peroxidase, and forms a dark to light brown precipitate inside the cells, whereby the intensity of the precipitate reflects the amounts of H_2_O_2_ in cells. *A. thaliana* seeds were pre-treated with 0, 100 and 200 µM Cu-chl while stratifying at 4 °C for three days. Seeds from each treatment were then grown in 0.5X liquid MS media in duplicates for 10 days. Finally, seedlings were exposed to 150 mM NaCl for three hours and collected for DAB staining.

**Figure 1:**
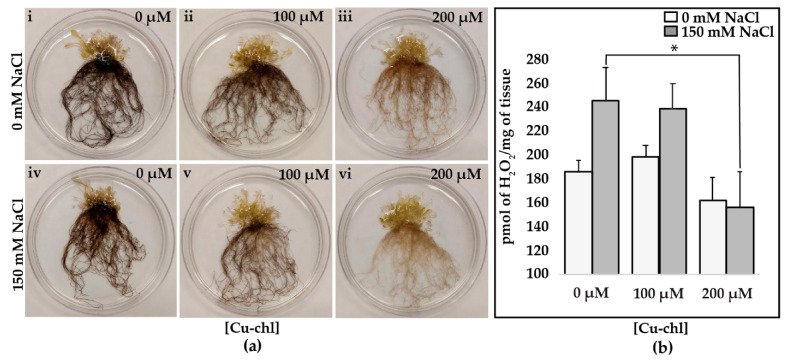
Copper chlorophyllin, Cu-chl, induced changes in cellular H_2_O_2_ accumulation under salt stress. Seedlings were grown in four biological replicates for each treatment under the same growth conditions. Finally, two of them were used for diaminobenzidine (DAB) assay and the other two for the Amplex^®^ Red assay, and the whole experiment was repeated twice: (**a**) DAB staining of *A. thaliana* seedlings in the absence (i–iii) of salt and after 3 h salt treatment (iv–vi). Note the lighter DAB staining in seedlings treated with 200 µM Cu-chl. (**b**) Quantification of H_2_O_2_ by Amplex^®^ Red assay. Mean ± SE was calculated from two biological and three technical replicates for each treatment. Asterisk indicates significant difference (*p* = 0.05) according to two-sample t-test.

We found that the pre-treatment of seeds with 200 µM Cu-chl reduced the level of H_2_O_2_ under salinity stress compared to untreated seeds as indicated by lighter DAB staining (Figure 1a). We measured quantitatively H_2_O_2_ content using Amplex^®^ Red assay, which showed a significant reduction of endogenous H_2_O_2_ in Cu-chl pretreated seedlings under salt stress (Figure 1b).

### 2.2. Cu-chl Protects Arabidopsis thaliana Seedlings from Herbicidal Damage by Reducing H_2_O_2_

**Figure 2:**
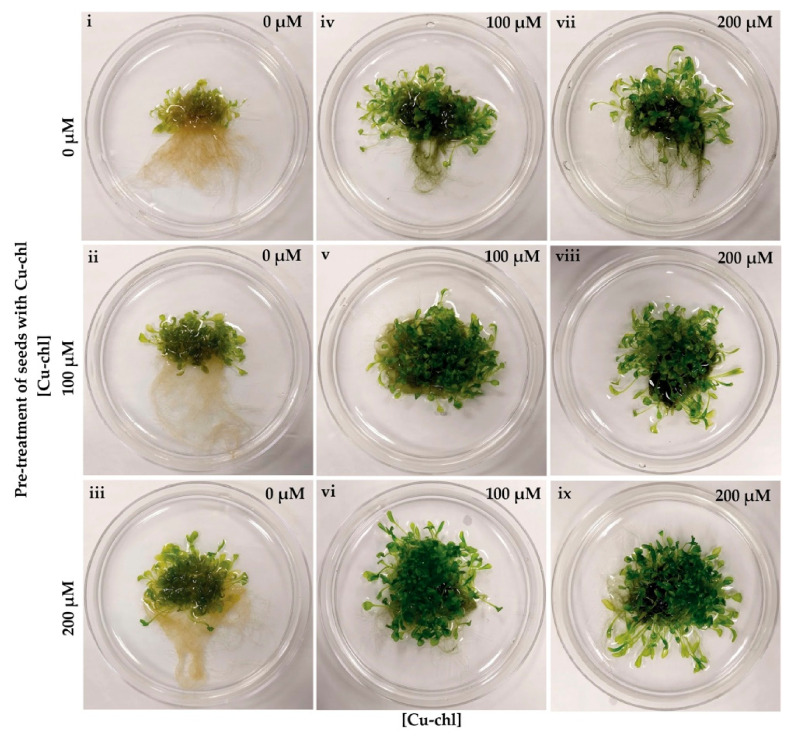
Paraquat induced phenotypes in Arabidopsis seedlings in the presence and absence of Cu-chl. Note bleaching on leaves, and stunted leaf growth (**i**) in the absence of Cu-chl. Cu-chl pre-treatment only (**ii**,**iii**), and pre-treatment and supplement (**iv**–**ix**) in the media showed improved growth and less bleaching of Arabidopsis seedlings.

**Figure 3:**
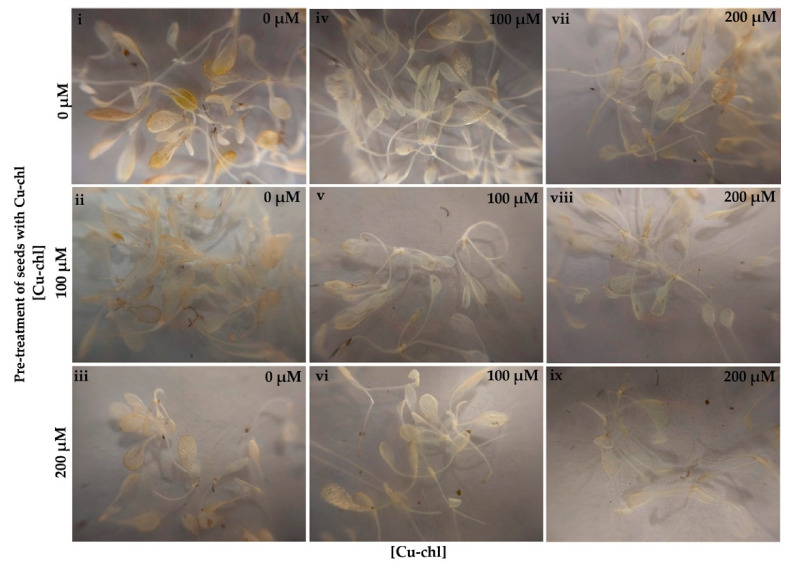
DAB staining of 2-week-old Arabidopsis seedlings pre-treated with Cu-chl and incubated with 100 nM paraquat for 3 h. Cu-chl was applied as seed treatment (concentrations indicated on the left) and in the growing media (concentrations indicated on the top right corner of each image) followed by paraquat treatment. Note the stronger DAB staining in leaves in the absence of Cu-chl treatment (i).

Accumulation of toxic levels of reactive oxygen species in tissues can be caused not only by various abiotic stresses, but also by the application of herbicides [38]. To determine whether Cu-chl has a protective effect against herbicide induced ROS, we used the ROS-generating herbicide, methyl viologen dichloride hydrate (paraquat), to induce oxidative stress in Arabidopsis seedlings [39]. Paraquat accepts electrons from photosystem I and transfers them to molecular oxygen, thereby producing destructive amounts of ROS. When treated with paraquat, Arabidopsis leaves displayed the typical paraquat induced phenotype and bleached under light, remaining small and yellow-brown in color (Figure 2i) [40], and DAB staining revealed higher peroxide accumulation in leaves (Figure 3i). While application of Cu-chl either to seeds or growth media reduced paraquat induced damage to leaf greening (Figure 2ii–ix) and reduced H_2_O_2_ accumulation in leaves (Figure 3ii–ix). In addition, pre-treatment and supplement of Cu-chl in the media reduced paraquat induced leaf growth inhibition (Figure 2), suggesting that Cu-chl can improve plant growth under stress.

### 2.3. RNA-Seq Reveals the Molecular Mechanism of Cu-chl

**Figure 4:**
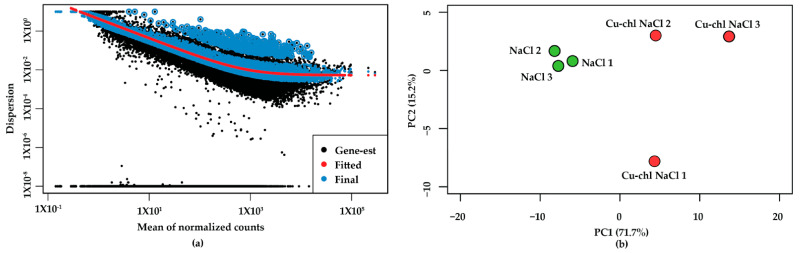
Distribution of samples and variation among the replicates and throughout the treatments: (**a**) dispersion/variation of each gene among the replicates. Black dot and blue circle designate, respectively, the mean of normalized counts and variation of a gene. Strongly clustered data points around the red line suggests that data are well distributed and fit for differential gene expression (DGE) analysis (**b**) principal component analysis (PCA) plot of relative distribution of biological replicates and the treatments. PCA1 and PCA2, respectively, denote the highest and second highest variation of samples among the treatments. All three replicates for NaCl showed strong grouping, where Cu-chl NaCls were dispersed on the plane. However, note the low percent of variation with PCA1, which indicates, there were no extreme outliers throughout the samples.

To investigate the H_2_O_2_ regulatory mechanism induced by Cu-chl, we conducted RNA-Seq under salinity stress with (Cu-chl NaCl in the following) and without treatment (NaCl in the following) of 1 mM Cu-chl. RNA-Seq data resulted in high quality reads across all three biological replicates in each treatment (Cu-chl NaCl, NaCl). Sequencing data and the statistics of their genomic alignment, considering average value of the replicates for each treatment is summarized in Table S1. For each of the treatments, the overall alignment percentage was reliable (more than 98%). We also assessed the variation of each gene between replicates by dispersion plot, which showed that data were clustered around the curve (red line, ideal fitted line for differential gene expression (DGE), with the dispersion decreasing with increasing mean expression levels (Figure 4a). This indicates that the data are a good fit for the DGE analysis [41]. Moreover, we estimated the variation among the replicates and throughout the treatments by principal component analysis (PCA) plot (Figure 4b). It showed strong grouping of replicates for NaCl, whereas data points for Cu-chl NaCl were dispersed on the plane. This implies that the expression of genes among the replicates of NaCl treatment is more convergent than Cu-chl NaCl. However, we considered all of them in the downstream analysis which makes it more conservative.

**Figure 5:**
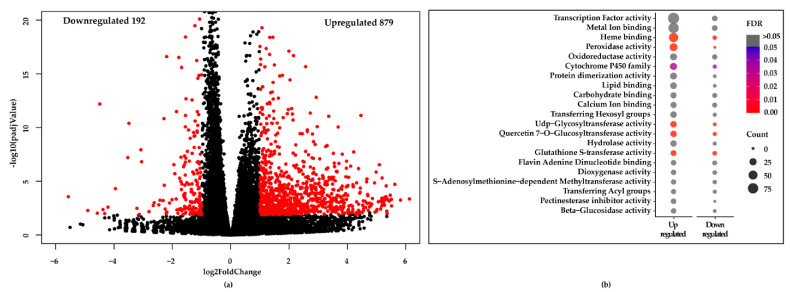
Differentially expressed genes and their corresponding molecular functions. (**a**) Volcano plot of significantly up and downregulated genes. X-axis and y-axis denote the log_2_FoldChange and -log10 of *padj* values, respectively; where log_2_FoldChange ≥ 1.0 and *padj* < 0.05 were considered as significant and indicated in red color. (**b**) Gene enrichment analysis of the significant genes. Color and bubble indicate the false discovery rate (FDR) and number of genes belong to each class, respectively.

Differentially expressed genes along with significantly up and downregulated genes (*padj* < 0.05 and log2FoldChange ≥ 1.0, indicated in red color) were determined using DESeq2 [41], and are shown as Volcano plot (Figure 5a). We found 879 genes were upregulated and 192 genes were downregulated in the Cu-chl NaCl compared to NaCl treatment (Figure 5a). Gene annotation for the significant genes was then carried out using DAVID bioinformatics resources v6.8 [42]. Top gene classes based on their molecular functions are shown as bubble plot, where color and size of the bubble, respectively indicate the False Discovery rate (FDR) and number of genes belong to each class (Figure 5b). Since many of them had higher FDR values (>0.05), we have manually checked each class and removed false positive associations.

Within the enriched cohort we found two predominant gene classes associated with H_2_O_2_ detoxification: 34 *peroxidases* and 16 *glutathione S-transferases* (*GSTs*), enriched with much lower FDR < 0.05 (Figure 5b, Table 1). Eleven of the 16 *GST* genes and all 34 *peroxidases* were more upregulated in the Cu-chl NaCl than NaCl. Among the 34 *peroxidases*, 26 were found to belong to the class III peroxidase super family (Table 1). Among the *GSTs* upregulated in Cu-chl NaCl, we found members of the *phi* (*GSTF*), *tau* (*GSTU*) and *lambda* (*GSTL*) classes, while members of the minor *GST* classes, *dehydroascorbate reductase* (*DHAR*s), and *tetrachlorohydroquinone dehalogenase* were not affected by Cu-chl (Table 1). In addition, we found 5 *Rboh* genes associated with H_2_O_2_ signaling and priming, were more upregulated in Cu-chl NaCl (Table 1) [43]. Interestingly, expression of other classes of antioxidant enzymes involved in H_2_O_2_ degradation/scavenging was not affected by treatment with Cu-chl (Table S2).

We also identified 33 transcription factors (TFs) that are reported to be involved in abiotic stress regulation. These include 9 *MYBs*, 5 *basic helix loop helix superfamily proteins* (*bHLH*), 7 *WRKYs*, 3 *NAC domain containing transcription factors*, 7 *Zinc finger proteins*, and 2 *Heat shock proteins* (Table 2). We also checked the differential expression of these peroxidases, glutathione S-transferases and TFs upon Cu-chl application under control conditions (without salt stress). We found that these genes were also induced by Cu-chl under control conditions, however less than when Cu-chl was applied with salt stress (Tables S3 and S4).

### 2.4. RNA-Seq Validation by Real-Time RT-PCR (qPCR)

**Figure 6:**
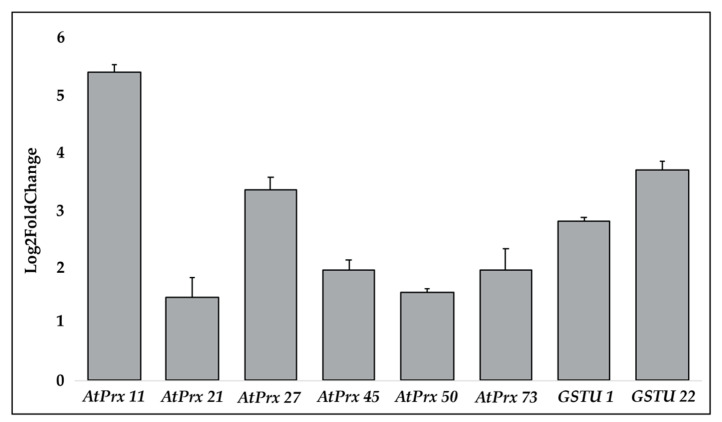
qPCR of class III peroxidase and glutathione S-transferase genes under salinity stress with and without Cu-chl treatment. Log2FoldChange (treated vs not treated) ≥1.0 was considered as significant upregulation, and all of them showed similar level of differential expression as RNA-Seq data. Log2FoldChange ± SE shown here are from three technical replicates of a biological sample.

To confirm the data obtained by RNA-Seq, the expression of 6 class III peroxidases (*AtPrx 11, AtPrx 21, AtPrx 27, AtPrx 45, AtPrx 50, and AtPrx 73*) and 2 *glutathione S-transferases* (*GSTU1, GSTU22*) were carried out by qPCR (primer sequences are in Table S5). qPCR results confirmed the data obtained by RNA-Seq, and showed all 6 class III *peroxidases*, and the two *glutathione S-transferases* were more upregulated in Cu-chl NaCl than NaCl treatment (Figure 6).

### 2.5. Effect of Cu-chl on Arabidopsis thaliana Growth

**Figure 7:**
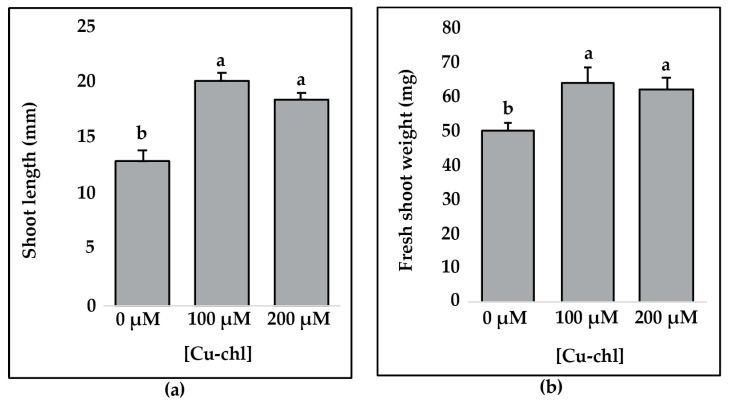
Effect of Cu-chl on growth and plant biomass: (**a**) shoot length of *A. thaliana* seedlings (n = 20, values are mean ± SE). (**b**) Fresh shoot weight (groups of four seedlings, n = 5) of the 20 seedlings measured in (**a**). Four randomly chosen seedlings were weighed together, different letters indicate significant difference between treatments according to Fisher’s least significant difference (LSD) test at *p* = 0.05.

To determine whether Cu-chl has beneficial effects on plant growth, we grew *A. thaliana* seeds in 0.5X liquid MS media supplemented with 100 and 200 µM Cu-chl and without Cu-chl for 14 days and measured the shoot length and fresh shoot weight. Both shoot length (Figure 7a) and weight (Figure 7b) were significantly higher with 100 and 200 µM Cu-chl treatment than in the control, suggesting that Cu-chl can improve plant growth.

## 3. Discussion

**Figure 8:**
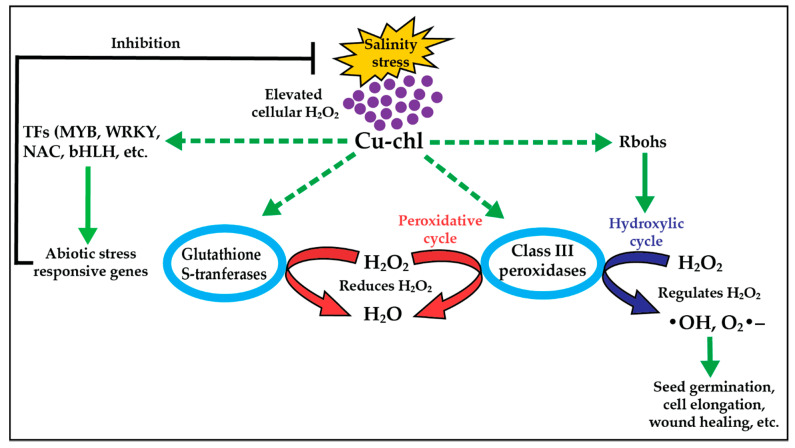
Mechanism of Cu-chl mediated oxidative stress regulation under salinity stress. Application of Cu-chl induces the expression of class III peroxidases, *Glutathione S-transferase*, Rbohs and TFs under salinity stress. Upon stimulation, class III peroxidases and glutathione S-transferases activate the peroxidative cycle and reduce H_2_O_2._ On the other hand, through Rbohs, Cu-chl activates the hydroxylic cycle, thereby producing •OH and O_2_•^–^ from H_2_O_2_ to help in cell growth. Solid and dashed green pointed lines, respectively, indicate the genes and mechanisms found in our RNA-Seq data and in previous studies.

To withstand salt stress, plants utilize various protective physiological, cellular and molecular mechanisms many of them geared towards reducing cellular concentrations of reactive oxygen species (ROS) (mainly hydrogen peroxide, H_2_O_2_) [29]. Exogenously applied biostimulants have been reported to be able to protect plants against stresses [30]. Our results indicate that pretreatment of Arabidopsis seedlings with Cu-chl can reduce cellular oxidative stress in salt or herbicide treated plants, primarily by decreasing cellular oxidative stress through modulating H_2_O_2_ levels. Although the precise molecular pathways through which Cu-chl maintains oxidative balance during stress are not known, genome wide analysis of RNA-seq data of salt stressed plants treated with Cu-chl suggests that Cu-chl may do that through upregulation of H_2_O_2_ detoxifying cellular pathways involving primarily class III peroxidases and Gluthathione S-transferases (Figure 8).

Class III peroxidases have been shown to scavenge H_2_O_2_ in vivo leading to increased ROS/H_2_O_2_ detoxification and osmotic adjustment, thereby improving tolerance to abiotic stresses. For instance, overexpression of *AtPrx 3* has been shown to increase tolerance under dehydration and salt stress, while its suppression gave dehydration and salt-sensitive phenotypes in *A. thaliana* [44]. In another study, *AtPrx 11* was found to be part of the complex network controlling cell expansion and cuticle deposition in response to osmotic stress, ABA and salt treatment [47]. *AtPrx 7* also has been reported to be involved in controlling the H_2_O_2_ concentration at the germination stage [98]. Functions of these peroxidases have, been investigated in other crop plants as well. In a study on rice, *OsPrx 24*, a class III peroxidase, was found to be regulated by a transcription factor complex and to function as a ROS scavenger to enhance tolerance against drought and salt stress [99]. Jin et al. showed overexpression of the class III peroxidase GsPRX 9 conferred soybean salt tolerance through mediation of the ROS regulatory network [100]. In transgenic Tobacco, heterologous expression of two class III peroxidases, *CrPrxs* from *Catharanthus roseus* exhibited increased tolerance to H_2_O_2_ treatment and improved germination rate under drought, salt and cold stress [101].

GSTs from all major classes have also been shown to be involved in the detoxification of toxic substances and attenuation of oxidative stresses [102]. In Arabidopsis, expression of two *GSTs* was found elevated in response to aluminum, cold, heat and metal stress, suggesting a common induction mechanism in response to the oxidative stresses [103]. Overexpression of a tobacco *GST* with *glutathione peroxidase* activity in transgenic tobacco showed increased glutathione-dependent peroxide scavenging that leads to reduced oxidative damage and enhanced tolerance to abiotic stresses [104]. They also found that cold or salt-stress treatments had less inhibitory effects on the growth of GST overexpressed transgenic lines. In barley leaves, a senescence-induced tau class GST has been suggested as an antioxidant, protecting senescing cells from ROS damages, and as an inducer that involves in secondary metabolism [105]. These studies support our data that upon Cu-chl treatment, class III peroxidases and GSTs act on reducing H_2_O_2_ under salinity stress. In addition, we found members of several transcription factor families, such as MYB, WRKY, NAC, bHLH, etc. that are known to play key roles in ROS signaling pathways in response to stresses, resulting in salt tolerance in Arabidopsis, rice and wheat [106].

This study exhibited that pretreatment of Arabidopsis seeds with Cu-chl was sufficient to reduce oxidative stress in salt or herbicide treated seedlings, suggesting treated plants have enhanced defensive capacity against salt stress. One possible scenario is that Cu-chl can activate defense signaling networks culminating in ROS scavenging through activation of genes involved in ROS signaling. We found 5 *NADPH/respiratory burst oxidases* (*Rbohs*) upregulated more in Cu-chl treated plants under stress. *Rbohs* are reported to be involved in ROS signaling, for example in barley, they have been marked as a hallmark of salt tolerant genotype, as their expression was increased significantly in a salt-tolerant mutant compared to the control after exposure to salt stress [106]. In Arabidopsis, overexpression of *RbohI* is reported to significantly improve the drought tolerance [61]. In another study on *Brassica campestris*, expression of *RbohA* and *RbohD* was found to be induced by different abiotic stressors like low temperature, salt and dehydration [55]. *Rbohs* are also reported to assist in class III peroxidases mediated induction of cellular processes like seed germination, cell elongation, lignification, wound healing, and plant senescence [107,108]. The O_2_•^–^ released during the oxidative cycle by Rboh can convert peroxidase into compound III, an oxygenated intermediate state of peroxidase. This compound consequently can catalyze the production of •OH from H_2_O_2_ in the cell wall and induce cell elongation [109]. We found enhanced seedlings growth by pre-treatment and supplement of Cu-chl in the media under both control (Figure 7) and stress conditions (Figure 2), which further supports this Cu-chl induced growth stimulatory pathways by class III peroxidases and Rbohs. Taken together, we propose a mechanism in which Cu-chl reduces H_2_O_2_ under salinity stress where class III peroxidases and GSTs function as the central molecules (Figure 8). Upon induction by Cu-chl, class III peroxidases and GSTs act by reducing the local concentration of H_2_O_2_ (peroxidative), and in association with Rbohs, class III peroxidases may favor plant growth by generating oxygen radicals (hydroxylic cycle) [110]. Cu-chl also stimulates TFs that can induce downstream salt responsive genes to alleviate stress.

In a paper published by Zhang et al. on tomato, Cu-chl was shown to alleviate oxidative stress caused by water deficit [37]. The availability of extensive genomic resources on Arabidopsis makes it the ideal host to investigate the functions and molecular mechanisms underpinning the role of Cu-chl in the mediation of plant defense under stress, but results obtained from Arabidopsis sometimes cannot be translated to crops. However, our data show that Cu-chl induced plant defense through upregulation of class III peroxidases, glutathione S-transferases and abiotic stress responsive transcription factors, which are highly conserved in plants. Further experiments should be done to see if the described mechanism is shared across plant species, and if the results seen in this study can be translated for field applications to other crops.

## 4. Materials and Methods

### 4.1. Seed Sterilization and Stratification

*A. thaliana* seeds (Columbia-0) were surface sterilized in 70% ethanol for 30 sec followed by 40% sodium hypochlorite for five min and rinsed four times in sterile water. For seeds that needed pre-treatment, Cu-chl was added at different concentrations (see paragraphs below) to 1 mL of sterile water directly to the seeds in an Eppendorf tube. Tubes containing seeds pre-treated or un-treated were then wrapped with aluminum foil and kept in the dark at 4 °C for three days.

### 4.2. H_2_O_2_ Accumulation Measurement via DAB (3,3′-Diaminobenzidin) and Amplex^®^ Red Assay under Salt Stress

After pre-treatment with 100 or 200 μM Cu-chl and three days incubation at 4 °C, four groups of 20 seeds per treatment (0, 100 and 200 μM Cu-chl) were placed in 10 mL of 0.5X liquid MS media in 12 separate Petri plates and grown in a shaking incubator at 21 °C and 20 rpm, maintaining 12 h light cycle for 10 days. Seedlings were then transferred to 10 mL of fresh 0.5 × liquid MS media, treated with 150 mM NaCl for three hours, washed with sterile distilled water and collected for DAB staining and Amplex^®^ Red assay. To qualitatively measure the level of H_2_O_2_ in the tissue, DAB assay was conducted using the SIGMA*FAST^TM^* DAB with Metal Enhancer kit (Sigma Aldrich, St. louis, MO, USA) following the user manual. In brief, four tablets from each of DAB/Cobalt and urea hydrogen peroxide were dissolved in 20 mL ultrapure MiliQ water (MilliporeSigma™ Milli-Q™ Ultrapure Water Systems, Thermo Fisher scientific, Waltham, MA, USA) by vortexing and poured in a 50 mL beaker. Seedlings from each treatment (two biological replicates of 20 seedlings/treatment) were dipped into the DAB solution and vacuum infiltrated for two minutes. After that, seedlings were placed in sterile distilled water in Petri plates and incubated in the dark overnight, when DAB is oxidized by H_2_O_2_ and forms dark-brown color. To observe color intensity, chlorophyll was removed by incubating the seedlings in 3:1 v:v, 90% ethanol:acetic acid at 70℃ for 10 min.

Quantification of H_2_O_2_ was carried out by using the Amplex^®^ Red hydrogen peroxide/peroxidase assay kit (Thermo Fisher Scientific, Waltham, MA, USA) on the two remaining biological replicates per each treatment. Standard curve was generated using H_2_O_2_ supplied in the kit (R^2^ = 0.98). Fifty mg of seedlings from each of the 6 plates (two out of the four biological replicates per each of the three treatments) were ground and diluted in 50 mM sodium phosphate buffer (pH = 7.4). Fluorescence intensity of three technical replicates for each of the six plates was measured by setting excitation at 560 nm and emission at 590 nm using SpectraMax^®^ i3x Multi-Mode Microplate Reader (Molecular Devices, San Jose, CA, USA). Finally, concentrations of H_2_O_2_ in the samples were calculated using the standard curve. Both DAB and Amplex^®^ Red assays were repeated twice.

### 4.3. H_2_O_2_ Accumulation Measurement under Herbicide Stress

Protective role of Cu-chl against herbicidal damage was determined by using a ROS-generating herbicide, methyl viologen dichloride hydrate (paraquat) (Sigma Aldrich, St. louis, MO, USA). After 0, 100 and 200 μM Cu-chl pre-treatment, followed by stratification at 4 °C, 12 petri plates of 20 seeds/plate for each treatment (0, 100 and 200 μM Cu-ch, total 36 plates) were grown in 10 mL of 0.5 × liquid MS media. Plates from each pre-treatment group were separated in three groups of four plates each. Four plates from each pre-treatment (12 plates) were grouped and supplemented with 0, 100 or 200 μM Cu-chl, so that each pre-treatment group was supplemented with all three post-treatments. After two weeks (growth conditions as in 4.2), 100 nM paraquat was applied in each Petri plate. Seedlings from two petri plates for each treatment were then collected for DAB assay three hours post paraquat application and the remaining two plates were left for 10 days to observe bleaching of the leaves.

### 4.4. Cu-chl and Salt Stress Application for RNA-Seq and qPCR

After stratification at 4 °C, 15 seeds/petri plate without any pre-treatment were grown in a shaking incubator maintaining the same growth conditions as mentioned above. Seedlings were then transferred to 10 mL of fresh 0.5 × liquid MS media containing either 100 μL DMSO (mock treatment) or 1 mM Cu-chl in DMSO. Seedlings were incubated for 24 h followed by two hours salt treatment with 100 mM NaCl. Cu-chl and NaCl concentrations, and duration of the treatment were different than the ones used in the assays above and were optimized based on preliminary experiments to reach maximum level of gene expression. Fifty mg of seedlings per plate and three biological replicates for each treatment (Control without any Cu-chl and NaCl, Cu-chl, NaCl and Cu-chl NaCl) were harvested for RNA extraction using Quick RNA Plant Miniprep kit (Zymo Research Corporation, Irvine, CA, USA).

### 4.5. Effect of Cu-chl on Seedling Growth

After pre-treatment with 100 and 200 μM Cu-chl, *A. thaliana* seedlings were grown for two weeks maintaining the same growth conditions as above. Twenty seedlings from each of the treatments (0, 100 and 200 μM Cu-chl) were randomly selected to measure shoot length using Image J and fresh shoot weight.

### 4.6. RNA Sequencing and Analysis

A uniquely barcoded library was made from each sample using the Illumina TruSeq Stranded mRNA Library kit (Illumina, Inc., San Diego, CA, USA) at the Genomic Core Facility, Penn State. An approximately equimolar pool of the libraries was made and sequenced on a NextSeq 550 high output system (Illumina, San Diego, CA, USA), which was set for 75 nt single read sequences. This provided about 30 million reads per sample. Sequences were analyzed using a series of bioinformatics tools in Bash and R background. In brief, raw reads for all of the replicates in each treatment were checked and preprocessed by Fastqc [111]. After checking the quality, reference genome (*A. thaliana*, TAIR 10) was used, and sequences were aligned and mapped against the genome by Hisat2 [112]. The aligned files were assembled and merged into one file by StringTie2 [113] and raw reads per sample were counted by featureCounts [114]. Finally, differential gene expression (DGE) analysis was carried out using the DESeq2 package in Bioconductor library [41] and differentially expressed genes throughout the treatments were functionally annotated by using DAVID bioinformatics resources v6.8 [42].

### 4.7. cDNA Synthesis and qPCR

Total RNA was extracted using Quick-RNA Plant Kit (Zymo Research, Irvine, CA, USA) the manufacturer’s instructions. 400 ng of total RNA was used for cDNA synthesis using the High-Capacity cDNA reverse transcription kit (Thermo Fisher Scientific, Waltham, MA, USA) according to the user manual.

qPCR was done using ssoAdvanced universal SYBR green super mix (Bio-Rad, Hercules, CA, USA) in BIO-RAD *CFX96 Touch™ Real-Time PCR Detection System* (Bio-Rad) following the manufacturer’s instructions. The reaction was set up in technical triplicates using Elongation factor 1 α as the internal control. Thermo cycling conditions were 95°C for 30 sec, followed by 39 cycles of 95 °C for 5 s, 60 °C for 30 s. A dissociation protocol with a gradient from 65 °C to 95 °C (5.0 secs and 0.5 °C ramp/cycle) was used for generating melting curves. Primers used for qPCR are listed in Table S5. Three biological replicates and three technical replicates were used for analysis. Finally, a software provided by Bio-Rad was used to analyze differential gene expression (DGE) using 2^-ΔΔCT^ method [115] and statistical analysis was done by two samples t-test using Minitab19 [116].

## Figures and Tables

**Table 1:** List of genes involved in H_2_O_2_ detoxification or signaling were more upregulated in Cu-chl NaCl compared to NaCl. AtPrx, arabidopsis thaliana Class III peroxidase; Trx, thioredoxin superfamily protein; Rboh, respiratory burst oxidase homolog/riboflavin synthase-like superfamily protein; Dox, alpha dioxygenase Tpx, thioredoxin-dependent peroxidase; GSTU, glutathione S-transferase class tau; GSTL, glutathione S-transferase lambda; GSTF, glutathione S-transferase class phi.

Function	Gene ID	Gene Name	log2Fold Change	Previously Reported	References
H_2_O_2_ detoxification	**Peroxidases**				
	** Class III peroxidases**				
	AT1G05260	*AtPrx3*	1.93	Cold inducible tolerance, Stamen abscission	[44,45]
	AT1G14550	*AtPrx5*	3.50		
	AT1G30870	*AtPrx7*	5.33	TNT treatment	[46]
	AT1G49570	*AtPrx10*	3.90		
	AT1G68850	*AtPrx11*	1.88	Cuticle metabolism regulation in response to abiotic stress	[47]
	AT2G18980	*AtPrx16*	2.75		
	AT2G37130	*AtPrx21*	1.43	Stamen abscission, aluminum stress	[45,48]
	AT2G38380	*AtPrx22*	2.02	potassium deficiency	[49]
	AT2G38390	*AtPrx23*	2.52		
	AT2G39040	*AtPrx24*	3.42		
	AT3G01190	*AtPrx27*	4.11	Aluminum stress, TNT treatment	[46,48]
	AT3G03670	*AtPrx28*	2.87		
	AT3G21770	*AtPrx30*	1.42	Cell elongation, Stamen abscission, Monolignin polymerization	[45,50,51]
	AT3G32980	*AtPrx32*	1.69	Cell elongation	[50]
	AT4G26010	*AtPrx44*	1.65		
	AT4G30170	*AtPrx45*	2.35	Cell elongation, aluminum stress, TNT treatment, Stamen abscission	[45,48,50,52]
	AT4G37520	*AtPrx50*	1.34	Low oxygen response, phosphate starvation, Stamen abscission	[45,53,54]
	AT5G06730	*AtPrx54*	2.07		
	AT5G14130	*AtPrx55*	2.82		
	AT5G15180	*AtPrx56*	1.42	Aluminum stress	[48]
	AT5G17820	*AtPrx57*	4.38	Arsenic stress, TNT treatment, cell elongation	[46,50,55]
	AT5G19890	*AtPrx59*	4.22	Aluminum stress, Mechanical stimulus	[48,51]
	AT5G24070	*AtPrx61*	3.46		
	AT5G64100	*AtPrx69*	2.67	Phosphate starvation, sulphur deficiency	[52,53]
	AT5G66390	*AtPrx72*	1.33	Cell elongation	[50]
	AT5G67400	*AtPrx73*	2.56	Aluminum stress	[48]
H_2_O_2_ detoxification and signaling	**Other peroxidases**				
	AT1G60740	*Trx*	4.66		
	AT5G07390	*RbohA*	2.94	Lateral root emergence, salinity and cold stress	[54,55]
	AT1G09090	*RbohB*	3.26	Nitrogen fixation, lateral root emergence	[56,57]
	AT5G51060	*RbohC*	1.99	Lateral root emergence, salinity and cold stress	[58,59]
	AT4G25090	*RbohG*	2.96	Lateral root emergence	[60]
	AT4G11230	*RbohI*	1.12	Drought stress	[61]
	AT3G01420	*Dox1*	2.93		
	AT1G65970	*Tpx2*	1.27		
H_2_O_2_ detoxification	**Glutathione S-transferase**				
	AT2G29490	*GSTU1*	2.79	Herbicide treatment, phytoremediation, oxidative stress response (SO_2_), salinity, drought and cold stress	[62,63,64,65,66]
	AT2G29480	*GSTU2*	2.63	Herbicide treatment, salinity and drought stress	[65,67]
	AT2G29470	*GSTU3*	2.64	Oxidative stress response (SO_2_)	[64]
	AT2G29460	*GSTU4*	1.76	Oxidative stress response (SO_2_), salinity	[64,68]
	AT2G29420	*GSTU7*	1.37	Seed germination, ABA response and osmotic stress	[69]
	AT3G09270	*GSTU8*	1.40	Cadmium treatment	[70]
	AT1G69920	*GSTU12*	1.68	Salinity stress	[71]
	AT1G27140	*GSTU14*	4.32		
	AT1G78340	*GSTU22*	2.89		
	AT1G17170	*GSTU24*	1.61	TNT treatment, herbicide treatment, phytoremediation, oxidative stress response (SO_2_)	[62,63,64]
	AT5G02780	*GSTL1*	1.27	Increased tolerance to salinity stress	[72]

**Table 2:** List of transcription factors (TFs) involved in abiotic stresses regulation and signaling that were more upregulated in Cu-chl NaCl compared to NaCl.

Gene ID	Gene Name	log2Fold Change	Previously Reported	References
**MYB containing domain**				
AT5G49620	*MYB 78*	4.44	Abiotic and biotic stress	[73]
AT1G74080	*MYB122*	3.18	Dehydration stress	[74]
AT1G79180	*MYB63*	2.52	Dehydration stress	[74]
AT5G54230	*MYB49*	2.39	Cadmium accumulation	[75]
AT1G09540	*MYB61*	2.17	Stomatal aperture	[76]
AT5G65790	*MYB68*	1.85	High temperature	[77]
AT1G48000	*MYB112*	1.75	Salinity and high light stress	[78]
AT4G34990	*MYB32*	1.35	Salinity stress	[79]
AT3G49690	*MYB84*	1.27	High temperature	[77]
**Basic helix-loop-helix DNA binding superfamily protein**				
AT4G21340	*bHLH*	4.04	Response to phytotoxicity	[80]
AT1G02340	*bHLH*	2.57	Dark induced senescence	[81]
AT4G29930	*bHLH*	1.67	Dehydration stress	[74]
AT1G10585	*bHLH*	1.46	Dehydration stress	[74]
AT5G51780	*bHLH*	1.11	Salinity stress	[82]
**WRKY DNA binding protein**				
AT1G68150	*AtWRKY09*	3.97	Abiotic stresses	[83]
AT5G15130	*AtWRKY72*	2.90	Abscisic acid signal	[84]
AT4G22070	*AtWRKY31*	2.70	Root growth, pathogen attack	[85]
AT5G13080	*AtWRKY75*	2.65	Leaf senescence	[86]
AT1G69810	*AtWRKY36*	1.61	UV responsive	[87]
AT1G30650	*AtWRKY14*	1.31	Abiotic stresses	[88]
AT3G01970	*AtWRKY45*	1.16	Dehydration stress tolerance	[89]
**NAC containing domain**				
AT3G18400	*ANAC058*	1.91	ABA mediated germination	[90]
AT1G01010	*ANAC001*	1.67	Dehydration stress	[74]
AT3G29035	*ANAC003*	1.34	Leaf senescence	[91]
**Zinc finger protein**				
AT1G67030	*AtZFP67*	3.98	ABA repressor	[92]
AT5G22890	*AtSTOP2 (C2HC ZFP)*	3.56	Aluminum and low pH	[93]
AT5G57520	*AtZFP2*	3.51	Salinity stress	[82]
AT1G10480	*AtZFP5*	3.31	Phosphate and potassium deficiency	[94]
AT1G68360	*AtGIS3 (C2HC ZFP)*	1.59	Cold stress	[95]
AT2G28200	*C2H2 ZFP*	1.12	Dehydration stress	[74]
AT2G19810	*AtOZF1(CCCH ZFP)*	1.08	Hydrogen peroxide, abscisic acid and salinity responsive	[96]
**Heat shock family protein**				
AT3G51910	*AtHSFA7A 2*	2.12	Heat shock response	[97]
AT2G26150	*AtHSFA2*	1.94	Heat shock response	[97]

## Data Availability

Data available in a publicly accessible repository.

## Supplementary Materials

The following are available online at https://www.mdpi.com/2223-7747/10/4/625/s1 Table S1. Summary of sequencing data and the statistics of their genomic mapping, Table S2. Genes that are part of the classical antioxidant system, and predominantly involved in H_2_O_2_ detoxification were not differentially expressed [117]. Table S3. Comparative differential expression of Peroxidases and Glutathione S-transferases with and without salt stress (control) upon Cu-chl application. Table S4. Comparative differential expression of abiotic stress responsive Transcription factors with and without salt stress (control) upon Cu-chl application. Table S5. Sequences of primers used in the study.
