# Supplementary material for: A Plant Based Modified Biostimulant (Copper Chlorophyllin), Mediates Defense Response in Arabidopsis thaliana under Salinity Stress
Source: Plants (Basel). 2021 Mar 25;10(4):625. doi: 10.3390/plants10040625 (PMC8064443; doi:10.3390/plants10040625)
Supplement: Supplementary file 1 [file plants-10-00625-s001.pdf]

Table S1. Summary of sequencing data and the statistics of their genomic mapping.

**Statistical analysis**	**Treatments**	
	**Cu-chl NaCl**	**NaCl**
Total reads	31666271.67	29237943.00
Unmapped sequences (No.)	373684.67	353123.33
Mapped sequences (aligned uniquely)	29579891.67	27970641.00
Mapped sequences (aligned >1)	1712695.33	914178.67
Overall alignment percentage (%)	98.82	98.79

Table S2. Genes that are part of the classical antioxidant system, and predominantly involved in H_2O_2 detoxification were not differentially expressed.

Gene ID	Gene Name	log2Fold Change	Previously Reported	References
	**Peroxidase**			
AT1G07890	Ascorbate peroxidase 1	-0.16		
AT3G09640	Ascorbate peroxidase 2	1.33		
AT4G35000	Ascorbate peroxidase 3	-0.35		
AT4G32320	Ascorbate peroxidase 6	0.05		
AT2G25080	Glutathione peroxidase 1	-0.09		
AT2G31570	Glutathione peroxidase 2	0.17		
AT2G43350	Glutathione peroxidase 3	-0.01		
AT3G63080	Glutathione peroxidase 5	0.28		[117]
AT4G11600	Glutathione peroxidase 6	0.39		
AT1G63460	Glutathione peroxidase 8	0.01	Involved in reducing H_2O_2	
	**Reductase**			
AT3G24170	Glutathione reductase 1	-0.29		
AT3G54660	Glutathione reductase 2	-0.35		
AT3G52880	Monodehydroascorbate reductase 1	-0.27		
AT3G09940	Monodehydroascorbate reductase 3	0.49		
AT3G27820	Monodehydroascorbate reductase 4	-0.01		
AT1G63940	Monodehydroascorbate reductase 6	-0.58	Involved in reducing H_2O_2	[117]
	**Dismutase**			
AT1G08830	Superoxide dismutase 1	0.56		
AT2G28190	Superoxide dismutase 2	0.46		
AT5G18100	Copper/Zinc Superoxide dismutase 3	-0.08	Involved in reducing H_2O_2	[117]
	**Glutathione S-transferase**			
AT1G19550	Dehydroascorbate reductase	-0.07		
AT1G19570	Dehydroascorbate reductase 5	-0.03		
AT1G75270	Dehydroascorbate reductase 2	0.51		
AT5G16710	Dehydroascorbate reductase 1	-0.53	Involved in reducing H_2O_2	[117]
	**Catalase**			
AT1G20630	Catalase 1	0.20	Involved in reducing H_2O_2	[117]

Table S3. Comparative differential expression of *Peroxidases* and *Glutathione S-transferases* with and without salt stress (control) upon Cu-chl application.

Gene ID	Gene Name	Log2Fold Change	
		Cu-chl NaCl vs NaCl	Cu-chl vs Control
**Class III peroxidases**			
AT1G05260	*AtPrx3*	1.93	0.47
AT1G14550	*AtPrx5*	3.50	1.9
AT1G30870	*AtPrx7*	5.33	1.23
AT1G49570	*AtPrx10*	3.90	3.65
AT1G68850	*AtPrx11*	1.88	0.99
AT2G18980	*AtPrx16*	2.75	0.61
AT2G37130	*AtPrx21*	1.43	1.75
AT2G38380	*AtPrx22*	2.02	0.57
AT2G38390	*AtPrx23*	2.52	0.81
AT2G39040	*AtPrx24*	3.42	0.26
AT3G01190	*AtPrx27*	4.11	1.68
AT3G03670	*AtPrx28*	2.87	2.46
AT3G21770	*AtPrx30*	1.42	0.99
AT3G32980	*AtPrx32*	1.69	0.26
AT4G26010	*AtPrx44*	1.65	0.36
AT4G30170	*AtPrx45*	2.35	1.77
AT4G37520	*AtPrx50*	1.34	1.88
AT5G06730	*AtPrx54*	2.07	1.34
AT5G14130	*AtPrx55*	2.82	0.86
AT5G15180	*AtPrx56*	1.42	1.01
AT5G17820	*AtPrx57*	4.38	1.21
AT5G19890	*AtPrx59*	4.22	1.7
AT5G24070	*AtPrx61*	3.46	-0.01
AT5G64100	*AtPrx69*	2.67	0.92
AT5G66390	*AtPrx72*	1.33	0.54
AT5G67400	*AtPrx73*	2.56	1.24
**Other peroxidases**			
AT1G60740	*Trx*	4.66	4.15
AT5G07390	*RbohA*	2.94	1.78
AT1G09090	*RbohB*	3.26	1.74
AT5G51060	*RbohC*	1.99	0.72
AT4G25090	*RbohG*	2.96	1
AT4G11230	*RbohI*	1.12	1.6
AT3G01420	*Dox1*	2.93	1.76
AT1G65970	*Tpx2*	1.27	1
**Glutathione S-transferase**			
AT2G29490	*GSTU1*	2.79	1.81
AT2G29480	*GSTU2*	2.63	2
AT2G29470	*GSTU3*	2.64	-1.13
AT2G29460	*GSTU4*	1.76	-0.05
AT2G29420	*GSTU7*	1.37	0.94
AT3G09270	*GSTU8*	1.40	1.33
AT1G69920	*GSTU12*	1.68	0.87
AT1G27140	*GSTU14*	4.32	-0.06
AT1G78340	*GSTU22*	2.89	1.21
AT1G17170	*GSTU24*	1.61	0.79
AT5G02780	*GSTL1*	1.27	1.15

Table S4. Comparative differential expression of abiotic stress responsive Transcription factors with and without salt stress (control) upon Cu-chl application.

Gene ID	Gene Name	Log2Fold Change	
		Cu-chl NaCl vs NaCl	Cu-chl vs Control
**MYB containing domain**			
AT5G49620	*MYB 78*	4.44	1.85
AT1G74080	*MYB122*	3.18	1.46
AT1G79180	*MYB63*	2.52	2.38
AT5G54230	*MYB49*	2.39	0.84
AT1G09540	*MYB61*	2.17	-0.4
AT5G65790	*MYB68*	1.85	0.88
AT1G48000	*MYB112*	1.75	1.06
AT4G34990	*MYB32*	1.35	0.7
AT3G49690	*MYB84*	1.27	0.99
**Basic helix-loop-helix DNA binding superfamily protein**			
AT4G21340	*bHLH*	4.04	-0.39
AT1G02340	*bHLH*	2.57	2.22
AT4G29930	*bHLH*	1.67	0.91
AT1G10585	*bHLH*	1.46	-0.25
AT5G51780	*bHLH*	1.11	0.78
**WRKY DNA binding protein**			
AT1G68150	*AtWRKY09*	3.97	1.81
AT5G15130	*AtWRKY72*	2.90	0.39
AT4G22070	*AtWRKY31*	2.70	2.83
AT5G13080	*AtWRKY75*	2.65	1.61
AT1G69810	*AtWRKY36*	1.61	1.01
AT1G30650	*AtWRKY14*	1.31	0.4
AT3G01970	*AtWRKY45*	1.16	1.27
**NAC containing domain**			
AT3G18400	*ANAC058*	1.91	2.45
AT1G01010	*ANAC001*	1.67	1.76
AT3G29035	*ANAC003*	1.34	0.4
**Zinc finger protein**			
AT1G67030	*AtZFP6 7*	3.98	2.05
AT5G22890	*AtSTOP2 (C2HC ZFP)*	3.56	0.72
AT5G57520	*AtZFP2*	3.51	-0.58
AT1G10480	*AtZFP5*	3.31	1.17
AT1G68360	*AtGIS3 (C2HC ZFP)*	1.59	1.17
AT2G28200	*C2H2 ZFP*	1.12	0.87
AT2G19810	*AtOZF1(CCCH ZFP)*	1.08	0.99
**Heat shock family protein**			
AT3G51910	*AtHSFA7A 2*	2.12	0.23
AT2G26150	*AtHSFA2*	1.94	-0.21

Table S5. Sequences of primers used in the study.

Gene ID	Gene Name	Primer Name	Primer Sequence (5'-3')
AT1G68850	*AtPrx 11*	AtPrx11 F	ACGAGCTTGCCACAACAAAC
		AtPrx11 R	GCGACATTGTGCTTTTCCGA
AT2G37130	*AtPrx 21*	AtPrx21 F	CGTCGCTCTCTTAGGTGCTC
		AtPrx21 R	GCGTAACTTGGGTCGAGAGT
AT3G01190	*AtPrx 27*	AtPrx27 F	AAAGGGCCTCAACGAGAAGG
		AtPrx27 R	GCTGTCTCCTTTTCCGGTGA
AT4G30170	*AtPrx 45*	AtPrx45 F	GCTGACATTTTGGCTCTCGC
		AtPrx45 R	AATTGGCTTTGAACGCTGGC
AT4G37520	*AtPrx 50*	AtPrx50 F	GGCCACTCGTGACGTCG
		AtPrx50 R	ATGCGGCAACTTCCCTCC
AT5G67400	*AtPrx 73*	AtPrx73 F	CCTGACACAACCACTGCTCA
		AtPrx73 R	GAGTAGCTGGGATGGTGACG
AT2G29490	*GSTU 1*	GSTU1 F	CCATGGCTCGATTTTGGGC
		GSTU1 R	GCAGTTCCCGAGTCTCTTCAA
AT1G78340	*GSTU 22*	GSTU22 F	GGGTTGATTTCGTGGACACC
		GSTU22 R	TGTCTCCAAGCTCAGTTTCAAGA
